# Supplementary material for: Determinants of left ventricular hypertrophy in a cohort attending a medical clinic: A comparative analysis stratified by sex
Source: PLOS Glob Public Health. 2026 May 12;6(5):e0006382. doi: 10.1371/journal.pgph.0006382 (PMC13166945; doi:10.1371/journal.pgph.0006382)
Supplement: Attachment — Submitted filename: Response to Reviewers.docx [file pgph.0006382.s002.docx]

Dear Dr. Chisompola,

We've checked your submission and before we can proceed, we need you to address the following issues:

Before we can continue with your submission, please upload copies of all the approval documents you received from your ethics committee to cover the entire range of the study period (i.e. the original approval document and any extension documents). Where ethics approval was obtained from more than one study location, please provide approval document(s) from all of the sites. If the original document is in another language, please also provide an English translation. Please note that if this documentation is not included when your manuscript is resubmitted, it may be rejected.

**Response**

Thank you for your request regarding submission of complete ethics approval documentation covering the full study period. We fully understand the critical importance of proper ethical documentation.

We confirm that ethical approval for this study was obtained from the Mulungushi University School of Medicine and Health Sciences Research Ethics Committee (SMHS-MU REC) (Reference No. SMHS-MU3-2023-005; approved 9 July 2023). Institutional authorization was also granted by Livingstone University Teaching Hospital management prior to commencement of the study.

The approval covered the entire duration of participant recruitment and data collection (October 1, 2023, to June 1, 2024). No extensions or amendments were required, as the study was completed within the originally approved timeframe.

This was a single-center study conducted exclusively at Livingstone University Teaching Hospital; therefore, no additional site-specific ethics approvals were required.

The original ethics approval letter from the Mulungushi University Research Ethics Committee is already uploaded together with other documents. Furthermore, Institutional authorization from Livingstone University Teaching Hospital was sought and granted by initiation of the study.

We confirmation that no extensions were issued or required.

All documentation is provided in English.

We have revised the ethical approval statement to make clearer

We trust that this satisfies the journal’s ethical documentation requirements. Please let us know if any additional clarification is needed.
