# Supplementary material for: Determinants of left ventricular hypertrophy in a cohort attending a medical clinic: A comparative analysis stratified by sex
Source: PLOS Glob Public Health. 2026 May 12;6(5):e0006382. doi: 10.1371/journal.pgph.0006382 (PMC13166945; doi:10.1371/journal.pgph.0006382)
Supplement: Attachment — Submitted filename: Response_to_Reviewers_auresp_1.docx [file pgph.0006382.s003.docx]

Dear Editor

We would like to thank you and the reviewers for the thorough review of our manuscript and the insightful comments and suggestions offered for improving the overall quality of the work. We have revised the paper accordingly and believe the current version of the paper meets the journal’s publication requirements.

Please find below our responses to the editor and reviewer comments, detailing the changes we have made to the paper.

**Journal Requirements:**

1. Please provide a detailed online Financial Disclosure statement. This is published with the article. It must therefore be completed in full sentences and contain the exact wording you wish to be published.

a) State the initials, alongside each funding source, of each author to receive each grant. For example: “This work was supported by the National Institutes of Health (####### to AM; ###### to CJ) and the National Science Foundation (###### to AM).”

**Response**

Thank you for this helpful suggestion. We have revised the funding statement to explicitly indicate the recipient author initials for each grant in accordance with journal guidelines.

b) State what role the funders took in the study. If the funders had no role in your study, please state: “The funders had no role in study design, data collection and analysis, decision to publish, or preparation of the manuscript.”

For more information, please go to our submission guidelines:

https://journals.plos.org/globalpublichealth/s/submission-guidelines#loc-financial-disclosure-statement

**Response**

Thank you for this important clarification. We have revised the funding statement to explicitly indicate that the funders had no role in the design, conduct, analysis, or reporting of the study, in accordance with journal requirements.

2. Please ensure that the funders and grant numbers match between the Financial Disclosure field and the Funding Information tab in your submission form. Note that the funders must be provided in the same order in both places as well.

**Response**

Thank you for highlighting this important point. We have carefully reviewed and harmonized the funding information across the manuscript and the submission system. The funder names, grant numbers, and their order are now fully consistent between the Financial Disclosure statement and the Funding Information section.

3. Please update your online Competing Interests statement. If you have no competing interests to declare, please state: "The authors have declared that no competing interests exist."

**Response**

Thank you for this clarification. We have revised the Competing Interests statement to align with the journal’s required wording.

4. Please ensure that you refer to Figure 2 in your text as, if accepted, production will need this reference to link the reader to the figure.

**Response**
Thank you for this observation. We have revised the Results section to explicitly cite Figure 2 in the main text to ensure proper linkage during production.

5. If the reviewer comments include a recommendation to cite specific previously published works, please review and evaluate these publications to determine whether they are relevant and should be cited. There is no requirement to cite these works unless the editor has indicated otherwise.

Additional Editor Comments (if provided):

[Note: HTML markup is below. Please do not edit.]

**Reviewers' comments:**

Please use the space provided to explain your answers to the questions above. You may also include additional comments for the author, including concerns about dual publication, research ethics, or publication ethics. (Please upload your review as an attachment if it exceeds 20,000 characters)

Reviewer #1: Very well presented manuscript regarding the prevalence of left ventricular hypertrophy and its correlation with several risk factors among the two sexes. Given that these data are more or less known in would suggest adding more information regarding the higher prevalence in women in the discussion section as well as strengthening the clinical implications section. Moreover, a central illustration may be added summarising the key findings in a scheme to.make the manuscript more appealing.

**Response**

We thank the reviewer for this thoughtful and constructive comment. In response, we have strengthened the Discussion to better contextualize the higher prevalence of LVH observed in women, with particular emphasis on sex-specific patterns of cardiac remodeling, the contribution of central adiposity and dyslipidaemia, and the possible influence of sex-specific LVH thresholds. We have also expanded the clinical implications section to highlight the need for sex-sensitive cardiovascular risk assessment in routine HIV and general medical care, including closer attention to blood pressure control, waist circumference, and metabolic risk profiling in women. In addition, we have prepared a proposed central illustration summarizing the main findings of the study, including the overall determinants of LVH and the distinct sex-stratified profiles observed in males and females.

Reviewer #2: I would like to thank the authors for submitting this interesting study addressing sex-specific determinants of left ventricular hypertrophy (LVH) in a sub-Saharan African cohort. The topic is clinically relevant, particularly given the growing burden of cardiovascular disease in this population and the importance of sex-based differences in risk stratification.

The study is strengthened by the use of echocardiographic assessment and the attempt to explore sex-stratified determinants, which provides additional insights beyond conventional analyses.

However, several issues should be addressed to improve the scientific rigor and interpretability of the manuscript:

1. Study design and causality

The cross-sectional design limits the ability to infer causal relationships between risk factors and LVH. While this limitation is briefly acknowledged, the discussion should more clearly distinguish association from causation and avoid any causal interpretation.

**Response**

We thank the reviewer for this important methodological observation. We have revised the Discussion to more explicitly emphasize the cross-sectional nature of the study and to clearly distinguish associations from causal relationships. We have carefully reviewed the manuscript to avoid causal language and now consistently interpret findings as associations rather than determinants in a causal sense

2. Sample size and model stability in stratified analyses

Although the total sample size is modest (n=333), the number of LVH cases in sex-stratified analyses appears relatively small, particularly in males. This is reflected by wide confidence intervals (e.g., heart failure in males), suggesting potential instability of the regression models. The authors should explicitly report events per variable (EPV) or justify model robustness.

**Response**

We thank the reviewer for this important methodological observation. We agree that the relatively small number of LVH cases in the sex-stratified analyses, particularly among males (n=15), may limit model stability and contribute to the wide confidence intervals observed.

We have now clarified this in the revised manuscript and explicitly acknowledge the limited events-per-variable (EPV) in the stratified models. Given the small number of outcome events, particularly in males, the number of covariates included in the final models was carefully restricted to avoid overfitting. Nonetheless, the EPV in the male subgroup remains below conventional recommendations, and the resulting estimates should therefore be interpreted with caution.

We have revised the Discussion to emphasize the potential for model instability and imprecision, and we now highlight the need for confirmation in larger, adequately powered studies.

3. Variable selection and potential overfitting

The manuscript mentions multivariable logistic regression, but the process for variable selection is not clearly described. If stepwise selection was used, this should be explicitly stated and justified, as it may increase the risk of overfitting.

**Response**

We thank the reviewer for this important comment. We have clarified the variable selection process in the Methods section. Variables included in the multivariable models were selected based on a combination of statistical significance in univariable analyses and clinical relevance informed by prior literature. Given the limited number of outcome events, particularly in sex-stratified analyses, the number of variables included in the final models was restricted to reduce the risk of overfitting. We did not rely on automated stepwise selection procedures, and model construction was guided by both epidemiological plausibility and events-per-variable considerations.

4. Definition and measurement of LVH

The criteria used for defining LVH by echocardiography should be clearly specified (e.g., indexed LV mass thresholds, guideline reference), as this is critical for reproducibility and comparison with other studies.

**Response**

We thank the reviewer for this important comment. We agree that explicit reporting of the echocardiographic definition of LVH is essential for reproducibility and comparability across studies. We have therefore revised the Methods section to clearly state that LVH was defined using left ventricular mass indexed to body surface area (LVMI, g/m²), derived from standard transthoracic echocardiographic measurements performed according to established echocardiography guidelines. We now explicitly report the sex-specific LVMI thresholds of >102 g/m^2^ for men and >88 g/m^2^ for women, in accordance with the recommendations of the American Society of Echocardiography and the European Association of Cardiovascular Imaging. We also clarify that echocardiographic acquisition followed a standardized ASE protocol. This revision improves methodological transparency and facilitates direct comparison with prior echocardiographic studies of LVH, including studies from HIV and African cohorts that have used guideline-based LV mass indexing approaches.

5. Confounding factors

Important confounders such as antihypertensive treatment, duration of hypertension, HIV-related factors (e.g., ART regimen, viral load), and renal function may influence LVH but are not clearly addressed. The authors should clarify whether these were considered or discuss them as limitations.

**Response**

We thank the reviewer for this important observation. We agree that several additional factors, including antihypertensive treatment, duration of hypertension, HIV-related characteristics, and renal function, may influence the development of LVH.

In our study, some HIV-related variables, including ART regimen and duration on ART, were collected and explored in univariable analyses; however, they were not retained in the final multivariable models due to lack of statistical significance and the need to limit the number of covariates in the context of a relatively small number of outcome events. Viral load was controlled by study design, as all participants living with HIV were required to have an undetectable viral load.

Data on duration of hypertension, detailed antihypertensive treatment, and renal function were not systematically available and therefore could not be included in the analysis. We have now expanded the Discussion to explicitly acknowledge these variables as potential confounders and a source of residual confounding. Future studies incorporating these factors are warranted to better characterize their contribution to LVH in this population.

6. Interpretation of sex differences

The finding that metabolic factors are more prominent in females while hemodynamic factors dominate in males is interesting but should be interpreted cautiously given the limited sample size and cross-sectional design. The discussion should avoid overgeneralization.

**Response**

We thank the reviewer for this important and thoughtful comment. We agree that the observed sex-specific patterns should be interpreted with caution given the cross-sectional design and the limited number of events in the stratified analyses, particularly among males.

We have revised the Discussion to avoid overgeneralization and to more clearly frame these findings as exploratory and hypothesis-generating. We now emphasize that while the observed differences are biologically plausible and consistent with existing literature, they require confirmation in larger, longitudinal studies before firm conclusions can be drawn.

7. Clarity and language

The manuscript would benefit from minor language editing for clarity and consistency.

**Response**

We thank the reviewer for this helpful suggestion. The manuscript has been carefully revised to improve clarity, grammar, and consistency of terminology throughout. We have standardized key terms, simplified sentence structure where appropriate, and corrected minor typographical and grammatical errors.

Overall, this is a meaningful study with potential clinical relevance. With the above revisions and clarification of methodological aspects, the manuscript would be significantly strengthened.

**Response**

We thank the reviewer for their positive and constructive assessment of our work. We have carefully addressed all comments and revised the manuscript accordingly, including clarification of methodological aspects, strengthening of the Discussion, and improvement of overall clarity. We believe these revisions have substantially improved the rigor and presentation of the manuscript.

Figure Resubmissions:

While revising your submission, we strongly recommend that you use PLOS’s NAAS tool (https://ngplosjournals.pagemajik.ai/artanalysis) to test your figure files. NAAS can convert your figure files to the TIFF file type and meet basic requirements (such as print size, resolution), or provide you with a report on issues that do not meet our requirements and that NAAS cannot fix.

After uploading your figures to PLOS’s NAAS tool - https://ngplosjournals.pagemajik.ai/artanalysis, NAAS will process the files provided and display the results in the "Uploaded Files" section of the page as the processing is complete. If the uploaded figures meet our requirements (or NAAS is able to fix the files to meet our requirements), the figure will be marked as "fixed" above. If NAAS is unable to fix the files, a red "failed" label will appear above. When NAAS has confirmed that the figure files meet our requirements, please download the file via the download option, and include these NAAS processed figure files when submitting your revised manuscript.

**Response**

Thank you for this guidance. We have processed all figure files using the PLOS NAAS tool and ensured that they meet the required specifications, including file format, resolution, and size. The revised manuscript includes the NAAS-processed figure files as requested.
