# Supplementary material for: Determinants of left ventricular hypertrophy in a cohort attending a medical clinic: A comparative analysis stratified by sex
Source: PLOS Glob Public Health. 2026 May 12;6(5):e0006382. doi: 10.1371/journal.pgph.0006382 (PMC13166945; doi:10.1371/journal.pgph.0006382)
Supplement: Attachment — Submitted filename: Response_to_Reviewers_auresp_2.docx [file pgph.0006382.s004.docx]

**Response to reviewers**

**Dear Editor**

**We would like to thank you and the reviewers for the thorough review of our manuscript and the insightful comments and suggestions offered for improving the overall quality of the work. We have revised the paper accordingly and believe the current version of the paper meets the journal’s publication requirements. Please find below our responses to the editor and reviewer comments, detailing the changes we have made to the paper.**

Reviewer #1: The authors have successfully revised their manuscript according to the reviewers comments. Having the majority of recommendations addressed i find the manuscript fit for publication.

Reviewer #2: Thank you for the revised manuscript. The study addresses a clinically relevant topic, and the revised version is improved. In particular, the authors have clarified the echocardiographic definition of LVH, better described the variable selection strategy for multivariable models, expanded the discussion of sex-specific findings, and more clearly acknowledged the limitations of the cross-sectional design and the limited events-per-variable in the sex-stratified analyses.

The main results appear internally consistent. The overall findings, including the associations of LVH with age, female sex, hypertension, and heart failure, are supported by the data presented. The sex-stratified analyses are also potentially informative, although they should remain interpreted as exploratory given the small number of LVH events in males and the resulting wide confidence intervals.

**I have only a few remaining issues before acceptance:**

Data availability: The current statement indicates that the data are available only upon request, subject to a data sharing agreement and IRB approval. Please ensure that this fully complies with the journal’s data sharing policy, or provide a more explicit justification for any restriction and clarify whether de-identified underlying data can be deposited or otherwise shared in a policy-compliant manner.

**Response**

We thank the reviewer for this important comment. We have revised the Data Availability Statement to clarify the ethical and privacy constraints governing the dataset and to explicitly state that de-identified data can be made available to qualified researchers upon reasonable request, subject to IRB approval and a formal data sharing agreement. This ensures compliance with both ethical requirements and the journal’s data sharing policy.

Final proofreading and formatting cleanup: Please carefully remove remaining track-changes/formatting artifacts and merged text fragments in the revised manuscript. A few visible wording and formatting issues remain and should be corrected in the final version.

**Response**

We appreciate the reviewer’s careful reading of the manuscript. In response, we have performed a comprehensive final proofreading and formatting review. All track changes and comments have been fully resolved and removed, and residual formatting artifacts, spacing inconsistencies, and merged text fragments have been corrected. The manuscript has also been standardized for formatting and presentation to ensure a clean and publication-ready version.

Reference list check: Please carefully review the references for duplication and consistency. At least one duplicate reference appears to remain in the current version.

**Response**

We thank the reviewer for this careful observation. We have thoroughly reviewed the reference list for duplication and formatting consistency. The duplicate reference has been removed, specifically the repeated citation to Canciello et al. (2024), “Sex-Related Differences in Left Ventricular Geometry Patterns in Patients With Arterial Hypertension,” JACC Advances, which appeared as references 19 and 20 in the previous version. The reference list has been revised accordingly, renumbered, and checked for consistency in style, punctuation, capitalization, journal formatting, and DOI presentation throughout.

Tone of interpretation: The revised discussion is improved, but the sex-specific conclusions should remain cautiously phrased given the cross-sectional design and model instability in subgroup analyses.
**Response**

We thank the reviewer for this important and insightful comment. We agree that, given the cross-sectional design and the limited number of events in sex-stratified analyses, the interpretation of sex-specific findings should be appropriately cautious. In response, we have revised the Discussion to moderate the tone of our conclusions, explicitly acknowledging the potential for model instability and the inability to infer causality. The language has been adjusted to emphasize that the observed sex-specific associations should be interpreted as exploratory and hypothesis-generating rather than definitive.

Overall, this is a meaningful and improved manuscript, and I believe it would be suitable for publication after these minor final corrections.

**Response**

We sincerely thank the reviewer for their positive and encouraging assessment of our manuscript. We appreciate the constructive feedback provided throughout the review process, which has significantly improved the clarity, rigor, and overall quality of the work. All remaining minor corrections have been carefully addressed, including revisions to wording, formatting, references, and interpretation. We believe the manuscript is now suitable for publication and meets the journal’s standards.
